# Duration of antibiotic treatment for common infections in English primary care: cross sectional analysis and comparison with guidelines

**DOI:** 10.1136/bmj.l440

**Published:** 2019-02-27

**Authors:** Koen B Pouwels, Susan Hopkins, Martin J Llewelyn, Ann Sarah Walker, Cliodna AM McNulty, Julie V Robotham

**Affiliations:** 1 Modelling and Economics Unit, National Infection Service, Public Health England, London NW9 5EQ, UK; 2 Department of Health Sciences, Global Health, University Medical Centre Groningen, University of Groningen, Groningen, Netherlands; 3 Health Economics Research Centre, Nuffield Department of Population Health, University of Oxford, Oxford, UK; 4 Healthcare-Associated Infection and Antimicrobial Resistance Department, National Infection Service, Public Health England, London, UK; 5 Directorate of Infection, Royal Free London NHS Foundation Trust, London, UK; 6 National Institute for Health Research Health Protection Research Unit on Healthcare Associated Infections and Antimicrobial Resistance, Oxford, UK; 7 Department of Global Health and Infection, Brighton and Sussex Medical School, Falmer, Brighton, UK; 8 Department of Microbiology and Infection, Brighton and Sussex University Hospitals NHS Trust, Brighton, UK; 9 Nuffield Department of Medicine, University of Oxford, UK; 10 Public Health England Primary Care Unit, Microbiology Department, Gloucestershire Royal Hospital, Gloucester, UK

## Abstract

**Objective:**

To evaluate the duration of prescriptions for antibiotic treatment for common infections in English primary care and to compare this with guideline recommendations.

**Design:**

Cross sectional study.

**Setting:**

General practices contributing to The Health Improvement Network database, 2013-15.

**Participants:**

931 015 consultations that resulted in an antibiotic prescription for one of several indications: acute sinusitis, acute sore throat, acute cough and bronchitis, pneumonia, acute exacerbation of chronic obstructive pulmonary disease (COPD), acute otitis media, acute cystitis, acute prostatitis, pyelonephritis, cellulitis, impetigo, scarlet fever, and gastroenteritis.

**Main outcome measures:**

The main outcomes were the percentage of antibiotic prescriptions with a duration exceeding the guideline recommendation and the total number of days beyond the recommended duration for each indication.

**Results:**

The most common reasons for antibiotics being prescribed were acute cough and bronchitis (386 972, 41.6% of the included consultations), acute sore throat (239 231, 25.7%), acute otitis media (83 054, 8.9%), and acute sinusitis (76 683, 8.2%). Antibiotic treatments for upper respiratory tract indications and acute cough and bronchitis accounted for more than two thirds of the total prescriptions considered, and 80% or more of these treatment courses exceeded guideline recommendations. Notable exceptions were acute sinusitis, where only 9.6% (95% confidence interval 9.4% to 9.9%) of prescriptions exceeded seven days and acute sore throat where only 2.1% (2.0% to 2.1%) exceeded 10 days (recent guidance recommends five days). More than half of the antibiotic prescriptions were for longer than guidelines recommend for acute cystitis among females (54.6%, 54.1% to 55.0%). The percentage of antibiotic prescriptions exceeding the recommended duration was lower for most non-respiratory infections. For the 931 015 included consultations resulting in antibiotic prescriptions, about 1.3 million days were beyond the durations recommended by guidelines.

**Conclusion:**

For most common infections treated in primary care, a substantial proportion of antibiotic prescriptions have durations exceeding those recommended in guidelines. Substantial reductions in antibiotic exposure can be accomplished by aligning antibiotic prescription durations with guidelines.

## Introduction

Many countries, including the United Kingdom, are trying to tackle antibiotic resistance by reducing unnecessary or inappropriate prescribing.^1^ ^2^ ^3^ The clear link between antibiotic prescribing and resistance at the individual^4^ ^5^ and population level^6^ indicates that reducing antibiotic prescribing might decrease—or should at least stabilise—levels of antibiotic resistance.^7^ ^8^ ^9^ Previous work has reported substantial over-prescribing of antibiotics in primary care in the UK^1^ ^10^ ^11^ and elsewhere.^3^ ^12^ Reducing unnecessary antibiotic use can be achieved by starting antibiotic treatments only when clearly indicated,^1^ ^11^ changing the choice of drug for specific conditions,^6^ or avoiding unnecessarily long durations of treatment.^13^ Strategies to control antibiotic overuse in primary care focus on the initial prescribing decision (whether to treat, and choice of agent) because it is not routinely possible to reassess patients who start antibiotics.^14^ Little attention has been given to the possibility of reducing antibiotic overuse in primary care by minimising unnecessarily prolonged treatment.^15^ Historically, general practitioners have been taught that antibiotic courses should be long enough to prevent the development of antibiotic resistance in the infection that is being treated, based on evidence of the emergence of resistance frequently being related to suboptimal dosing of penicillins in the treatment of *Streptococcus pneumoniae*.^16^ However, current concerns primarily relate to the development of resistance in common commensal bacteria, rather than in the ones causing the infections, where there is increasing evidence that the opposite is true—the longer the exposure to antibiotic the greater the development of antibiotic resistance, which then leads to a greater risk of resistance in subsequent infections.^17^ ^18^ Overuse of antibiotics not only contributes to increased antibiotic resistance levels but also puts patients at risk of side effects. Common side effects include diarrhoea, rash, and candidiasis.^19^ ^20^ Cumulative exposure to antibiotics has been identified as a major risk factor for *Clostridium difficile* infection, highlighting the need to reduce the duration of treatment where possible.^21^ ^22^ Although less common, other serious side effects are important reasons to minimise exposure to particular antibiotics. For example, macrolides are associated with an increased risk of serious ventricular arrhythmias and increased risk of sudden cardiac death.^23^ Moreover, the US Food and Drug Administration recently strengthened its warning about fluoroquinolones potentially causing substantial decreases in blood sugar levels and mental health side effects.^24^

Evidence about the contribution of excessive treatment duration to antibiotic overuse in primary care is limited. A recent study from the US showed that more than two thirds of antibiotic courses for acute sinusitis in adults were 10 days or longer, whereas the Infectious Disease Society of America recommends treatment of uncomplicated cases for five to seven days.^25^ A small study from the Manitoba region in Canada, that focused on urinary tract infection, pharyngitis, skin or soft tissue infections, and pneumonia estimated that 15% of prescriptions with the appropriate antibiotic were for treatments longer than guideline based recommendations.^26^

An up-to-date picture on prescribed antibiotic durations for common infections in English primary care is lacking. Such an overview is especially relevant given the increasing evidence from randomised controlled trials and meta-analyses that shorter antibiotic courses clear infection comparable to longer courses, while minimising selection, proliferation, and spread of antibiotic resistant bacteria and the likelihood of side effects from antibiotic use.^18^ ^27^ ^28^

We therefore assessed the extent to which durations of antibiotic courses prescribed for common infections in English primary care are in line with relevant guidelines. If substantial proportions of antibiotic prescriptions are longer than recommended, this would indicate that there is potential to safely reduce total antibiotic use simply by better application of guidelines in clinical practice.

## Methods

Data were obtained from The Health Improvement Network (THIN), a primary care electronic database that contains anonymised data on patients, practices, and consultations and is representative of the general UK population, with consultation and prescribing rates similar to national data.^29^ We used the same data extract that was previously used to evaluate which antibiotics are prescribed for different conditions in England.^29^ This extract was limited to English practices that participated in THIN and provided data for at least one full calendar year between 1 January 2013 and 31 December 2015. The prescription-diagnosis linkage is described in detail elsewhere.^29^ For the current analyses, we included only prescriptions for oral antibiotics linked to one of several indications: acute sinusitis, acute sore throat, acute cough and bronchitis, pneumonia, acute exacerbation of chronic obstructive pulmonary disease (COPD), acute otitis media, acute cystitis, acute prostatitis, pyelonephritis, cellulitis, impetigo, scarlet fever, and gastroenteritis. We excluded chronic and recurrent conditions, operationalised by excluding consultations explicitly coded as such and consultations where patients received antibiotics for a condition of the same body system (respiratory, urinary, gastrointestinal, or skin) in the 30 days before the current antibiotic prescription. In addition, we excluded prescriptions that were explicitly coded as a repeat prescription or were part of a sequence of prescriptions where the same antibiotic was prescribed every month for at least six months or covered more than 162 exposure days over a period of 180 days.^29^ Actual durations of antibiotic prescriptions for the 13 indications considered were compared with durations recommended in English guidance provided by Public Health England (PHE)—using the PHE 2013 guidance for the main analysis (see table 1 and supplementary table S1): acute sinusitis,^30^ ^31^ acute sore throat,^31^ ^32^ acute cough and bronchitis,^31^ ^33^ pneumonia,^31^ ^34^ acute exacerbation of COPD,^31^ ^35^ acute otitis media,^31^ ^36^ acute cystitis,^31^ ^37^ acute prostatitis,^31^ ^38^ pyelonephritis,^31^ ^39^ cellulitis,^31^ ^40^ impetigo,^31^ ^41^ scarlet fever,^31^ ^42^ and gastroenteritis.^31^ ^43^

**Table 1:** Antibiotic treatment durations for first line antibiotics recommended by Public Health England (PHE) guidance during 2013-15

Indications	PHE recommendation for antibiotic prescription duration of first line antibiotics, 2013-15
Acute sinusitis	7 days
Acute sore throat	10 days
Acute cough and bronchitis	5 days
Community acquired pneumonia and CRB65=0	7 days
Community acquired pneumonia and CRB65=1	7-10 days
Acute exacerbation of COPD	5 days
Acute otitis media	5 days
Acute cystitis: non-pregnant females	3 days
Acute cystitis: males	7 days
Acute prostatitis	28 days
Pyelonephritis	7 days, except coamoxiclav, which is 14 days
Cellulitis	7 days, and continue for further 7 days if slow response
Impetigo	7 days
Scarlet fever	10 days*
Gastroenteritis	5-7 days

CRB65=confusion, respiratory rate, blood pressure, 65 years and older; COPD=chronic obstructive pulmonary disease.

* Not in PHE 2013 guidance; based on National Institute for Health and Care Excellence Clinical Knowledge Summaries guidance and PHE 2017 guidance.

### Statistical analysis

For each indication we calculated the proportion of prescriptions longer than the recommended duration separately for children (<16 years) and for people aged 16 years or older, and by antibiotic where guidelines recommended different durations for particular drugs. Because guidelines recommend longer antibiotic courses to treat acute cystitis in males than females,^31^ ^37^ we performed separate analyses for males and females for this condition. The cause of acute prostatitis in young men is often different^44^ from that in older men (sexually transmitted organisms versus mostly Enterobacteriaceae, respectively), and prescribers might fear chronic prostatitis more in older men. Therefore we evaluated three age groups for acute prostatitis: <35, 35-65, and >65 years old. In addition, for all conditions we performed a secondary analysis restricting to healthy patients, defined as those with no chronic kidney disease, COPD, asthma, coronary heart disease, immunosuppressive disease, or use of immunosuppressive drugs, systemic corticosteroids, or inhaled corticosteroids.^11^ ^45^

We used multiple imputations through chained equations using sequential regression trees to impute duration data that were missing in 10-20% of the consultations (see supplementary file 2 for more details).^46^ Confidence intervals were calculated using multiple imputation Wilson intervals, which have better properties than the usual multiple imputation confidence intervals, in particular always being bounded by zero and one.^47^ In sensitivity analysis, we restricted the analysis to data with no missing values.

In addition, we calculated the total number of excess antibiotic days, defined as the total number of days beyond the recommended duration in the guidelines.

All analyses were performed using R version 3.4.3 (packages: “dplyr,” “ggplot2,” “mice,” “nlme”).

### Patient and public involvement

No patients were involved in setting the research question or the outcome measures, nor were they involved in developing plans for design or implementation of the study. No patients were asked to advise on interpretation or writing up of results. Results will be disseminated to relevant patient communities through news media.

## Results

**Fig 1:**
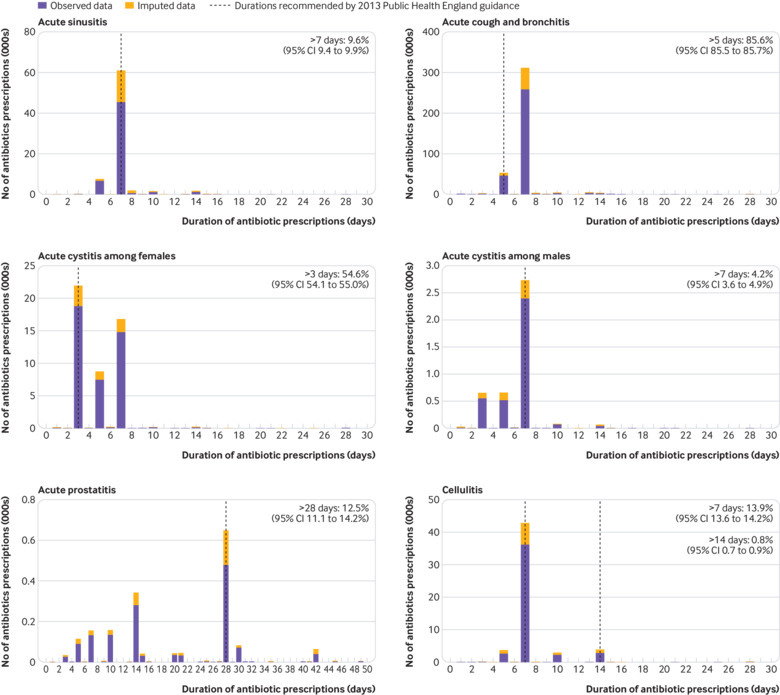
Durations of antibiotic prescriptions for various indications

Between 2013 and 2015, 931 015 consultations for the 13 included indications led to antibiotic prescriptions. This subset—which focused on common conditions, but excluded chronic and recurrent cases, repeat prescriptions, and antibiotic prophylaxis—covered about 20% of total antibiotics being prescribed (for any condition) during the study period. The most common indications were acute cough and bronchitis (386 972, 41.6% of the included consultations), acute sore throat (239 231, 25.7%), acute otitis media (83 054, 8.9%), acute sinusitis (76 683, 8.2%), cellulitis (54 610, 5.9%), and acute cystitis (53 010, 5.7%). Durations of antibiotic treatment for the included indications showed poor guideline adherence for several indications (fig 1 and supplementary figs S1-S8).

For all conditions grouped together, about 1.3 million days beyond the durations recommended by guidelines (table 2), which remained the same during 2013-15.

**Table 2:** Percentage of antibiotics with a duration exceeding guideline recommendations for all patients and antibiotics

Recommended treatment duration (days) by condition	No with condition	Antibiotic prescriptions with duration exceeding recommendations			Excess days (% of total days)
		No	% (95% CI)		
Acute sinusitis:					
7	76 683	7384	9.6 (9.4 to 9.9)		39 422 (7.1)
Acute sore throat:					
5*	239 231	200 520	83.8 (83.7 to 84.0)		640 381 (35.0)
10*	239 231	4972	2.1 (2.0 to 2.1)		72 001 (3.9)
Acute cough and bronchitis:					
5	386 972	331 257	85.6 (85.5 to 85.7)		805 051 (29.5)
Community acquired pneumonia:					
7†	952	77	8.1 (6.4 to 10.1)		744 (10.3)
10†	952	32	3.4 (2.3 to 4.9)		535 (7.4)
Acute COPD exacerbation:					
5	12 067	10 742	89.0 (88.4 to 89.6)		26 732 (30.8)
Acute otitis media:					
5	83 054	71 750	86.4 (86.1 to 86.6)		193 262 (31.9)
Acute cystitis: females:					
3	48 734	26 591	54.6 (54.1 to 55.0)		99 321 (40.5)
Acute cystitis: males:					
7	4276	181	4.2 (3.6 to 4.9)		1541 (5.6)
Acute prostatitis:					
28	1838	231	12.5 (11.1 to 14.2)		2806 (7.3)
Pyelonephritis:					
7‡	1948	351	18.0 (16.3 to 19.9)		2135 (14.1)
14‡	1948	7	0.3 (0.1 to 0.9)		269 (1.8)
Cellulitis:					
7§	54 610	7610	13.9 (13.6 to 14.2)		47 063 (11.2)
14§	54 610	424	0.8 (0.7 to 0.9)		6719 (1.6)
Impetigo:					
7	16 599	1646	9.9 (9.4 to 10.4)		13 948 (11.8)
Scarlet fever:					
5¶	2350	1887	80.3 (78.5 to 82.0)		9808 (45.7)
10¶	2350	157	6.7 (5.7 to 7.9)		1493 (7.0)
Gastroenteritis:					
5**	1701	906	53.3 (50.8 to 55.8)		3071 (27.3)
7**	1701	108	6.3 (5.2 to 7.7)		1260 (11.2)

COPD=chronic obstructive pulmonary disease.

* When antibiotics are indicated, Public Health England (PHE) 2013 guidance recommends 10 days when using penicillin V and five days when using clarithromycin for sore throat.

† PHE 2013 guidance recommends seven days for patients with a CRB65 (confusion, respiratory rate, blood pressure, ≥65 years) score of 0 and 7-10 days in patients with a CRB65 score of 1.

‡ PHE 2013 guidance recommends seven days when treating pyelonephritis with ciprofloxacin and 14 days when using coamoxiclav.

§ For patients with cellulitis, PHE 2013 guidance recommends starting with seven days and in case of a slow response continuing for another seven days. The Clinical Knowledge Summaries (CKS) guidance (last revised July 2015) recommends a treatment duration of seven days, except for patients with known lymphoedema for whom antibiotics should be continued ≥14 days beyond first observation of clinical response.

¶ Scarlet fever was not included in the PHE 2013 guidance. PHE 2017 guidance and CKS guidance recommend 10 days of treatment when using penicillin V. The CKS guidance recommends treating children with amoxicillin for 10 days in case penicillin V is not deemed suitable. For other antibiotics (clarithromycin or azithromycin) guidelines recommend a treatment duration of five days.

** Where patients with gastroenteritis are systemically unwell and campylobacter infection is suspected, a duration of 5-7 days is recommended by PHE 2013 guidance.

Most of the excess days were due to respiratory indications (table 2). Antibiotic treatments for respiratory indications, including otitis media, accounted for more than two thirds of the total prescriptions considered, and 80% or more of these treatment courses exceeded guideline recommendations (table 2). A notable exception was acute sinusitis, for which only 9.6% (95% confidence interval 9.4% to 9.9%) of prescriptions were longer than the seven days recommended by the PHE 2013 guidance. For some indications, guidelines recommend longer durations for patients who are more unwell, such as those with pneumonia and a CRB65 (confusion, respiratory rate, blood pressure, 65 years and older) score of 1 or 2, or a range of appropriate durations (supplementary table S1). A much smaller proportion of patients received antibiotic prescriptions exceeding these upper boundaries (table 2 and supplementary table S3).

When restricting to antibiotic prescriptions beyond the recommended durations, the median number of days beyond the guideline recommendation was 2 (5th-95th centile 2-3 days) for acute cough and bronchitis, 2 (2-8) days for acute otitis media, and 3 (1-7) days for acute sinusitis. This was 7 (3-8) days for cellulitis (using seven days as threshold), and for acute cystitis 4 (2-4) days for females and 7 (3-32) days for males. In general, the tendency was to write prescriptions with a duration of five or seven days or multiples thereof (fig 1 and supplementary figs S1-S8). The peak at seven days, however, tended to be higher than for five days, even for conditions where a duration of five days is recommended. For conditions where guidelines recommend longer durations, the percentage of prescriptions beyond the recommendation was substantially lower than for conditions where guidelines recommend a relatively short duration.

For respiratory tract indications the total number of days of prescribed antibiotics beyond the recommended duration (approximately 1.1 million excess days in total) comprised a substantial proportion of the total number of days of antibiotics prescribed for these indications—for example, 29.5% for acute cough and bronchitis (table 2).

Fewer prescriptions exceeded recommended durations for non-respiratory tract indications, but still more than half of the antibiotic prescriptions were for longer than guidelines recommend for acute cystitis among females (54.6%, 95% confidence interval 54.1% to 55.0%) (table 2). Although guidelines recommend a shorter duration for fosfomycin,^31^ this did not affect our comparisons because this antibiotic was only used in two patients with acute cystitis. For acute prostatitis, the percentage of antibiotics prescribed for longer than the recommended duration was 12.5% (11.1% to 14.2%) using the recommendation of treatment for 28 days (table 2). When antibiotics are indicated for gastroenteritis, guidelines recommend prescribing antibiotics for 5-7 days. Setting the threshold at five days, 53.3% (50.8% to 55.8%) of prescriptions were for longer than guideline recommendations, whereas this percentage was 6.3% (5.2% to 7.7%) when using the seven day threshold (table 2).

The percentage of antibiotic prescriptions for which the actual duration was beyond the recommendation was much lower for pyelonephritis (18.0%, 16.3% to 19.9%), acute cystitis among males (4.2%, 3.6% to 4.9%), impetigo (9.9%, 9.4% to 10.4%), and cellulitis (13.9%, 13.6% to 14.2%), but this still translated into 155 238 excess antibiotic days (table 2).

For acute prostatitis and acute cystitis among males, there appeared to be substantial under-treatment, with a substantial number of courses shorter than the guideline recommendations, with 52.3% below 28 days and 26.0% below 14 days for acute prostatitis and 31.8% below seven days for acute cystitis among males (fig 1). In addition, some under-treatment was observed when using trimethoprim for pyelonephritis, antibiotics for impetigo, and penicillin V for scarlet fever (supplementary figs S5, S6, and S7, respectively).

There was no clear tendency to prescribe longer courses for children (<16 years) compared with adults (≥16 years) (supplementary table S2). Nevertheless, the percentage of prescriptions that were longer than recommended was higher among younger patients for acute cough and bronchitis (89.0% *v* 84.7%), acute otitis media (89.9% *v* 78.6%), impetigo (12.1% *v* 6.4%), pyelonephritis (27.2% *v* 17.7%), and gastroenteritis (70.7% *v* 51.0%).

Similar results were obtained when restricting the analyses to patients without relevant comorbidities (table 2 and table 3). After excluding patients with comorbidities and previous use of immunosuppressive drugs or inhaled or systemic corticosteroids, the percentage of prescriptions that were longer than recommended differed by 2% or less, with the estimates based on all patients.

**Table 3:** Percentage of antibiotics with a duration exceeding guideline recommendations for patients without comorbidities or previous use of immunosuppressive drugs, inhaled corticosteroids, or systemic corticosteroids

Recommended treatment duration for comorbidities when antibiotics indicated (days)	No of patients	Antibiotic prescriptions with duration exceeding recommendations	
		No of prescriptions	% (95% CI)
Acute sinusitis:			
7	51 206	4701	9.2 (8.9 to 9.5)
Acute sore throat:			
5	188 708	156 722	83.1 (82.9 to 83.2)
10	188 708	4114	2.2 (2.1 to 2.3)
Acute cough and bronchitis:			
5	204 867	175 193	85.5 (85.4 to 85.7)
Community acquired pneumonia:			
7	434	30	6.8 (4.7 to 9.9)
10	434	11	2.6 (1.3 to 4.9)
Acute COPD exacerbation*:			
5	5737	5116	89.2 (88.3 to 90.0)
Acute otitis media:			
5	69 217	60 170	86.9 (86.7 to 87.2)
Acute cystitis females:			
3	31 794	17 008	53.5 (52.9 to 54.0)
Acute prostatitis:			
28	1240	159	12.8 (11.0 to 14.8)
Pyelonephritis:			
7	1347	254	18.9 (16.8 to 21.2)
14	1347	3	0.2 (0.06 to 0.9)
Cellulitis:			
7	26 041	3217	12.4 (12.0 to 12.8)
14	26 041	206	0.8 (0.7 to 0.9)
Impetigo:			
7	13 457	1398	10.4 (9.9 to 10.9)
Scarlet fever			
5	2081	1673	80.4 (78.5 to 82.2)
10	2081	142	6.8 (5.8 to 8.1)
Gastroenteritis:			
5	1193	628	52.7 (49.7 to 55.7)
7	1193	78	6.5 (5.1 to 8.3)

COPD=chronic obstructive pulmonary disease.

* Only considered other comorbidities than COPD and ignored corticosteroid use.

When restricting the analyses to antibiotics only mentioned in the guidelines, results were generally similar to the analyses including all antibiotics (supplementary table S3). A notable exception was gastroenteritis. The percentage of antibiotic prescriptions longer than guidelines recommended became lower for gastroenteritis when only considering antibiotics mentioned in the guidelines (40.0% *v* 53.3%, using a threshold of five days). In addition, for sore throat, pyelonephritis, and scarlet fever, longer prescriptions are recommended for specific antibiotics. In these cases, observed durations less frequently exceeded durations recommended in the guidelines compared with other antibiotics prescribed for the same condition (supplementary table S3). For most conditions, 75% or more of the prescribed antibiotics were mentioned in the guidelines for treatment of that condition (supplementary table S3). A notable exception was gastroenteritis, where only 12.8% of the patients were treated with the recommended antibiotic.

Sensitivity analysis restricting the analysis to data with no missing values gave similar results to the main analysis (supplementary table S4).

## Discussion

For most common infections treated in primary care, a substantial proportion of antibiotic prescriptions have durations exceeding those recommended in guidelines. This is most noticeable for most respiratory indications and acute cystitis among females. If all patients receiving antibiotics for the included indications received treatment for durations recommended by guidelines over the study period (three years in about 4.4% of the English population; included prescriptions captured around 20% of total antibiotic prescriptions in the included practices during the study period), this would equate to 1.1 million fewer days of antibiotics for respiratory tract indications and 100 000 fewer days for acute cystitis among females. Our findings indicate substantial scope for reducing antibiotic prescribing through better adherence to recommended durations of antibiotic treatment. In contrast, the duration of antibiotic prescriptions for pyelonephritis, acute cystitis among men, impetigo, and cellulitis generally did not exceed guideline recommendations, with only 4-18% of prescriptions exceeding them.

### Comparison with other studies

This study assessed the extent to which the duration of antibiotic prescriptions, for a wide range of common infections, follow guidelines in English primary care. In contrast with a recent US study only focusing on acute sinusitis,^25^ we were able to account for comorbidities and previous use of drugs that potentially influence the duration of prescriptions. We found similar results when restricting to healthy patients without comorbidities or past use of immunosuppressive drugs or corticosteroids, suggesting that comorbidities do not play a major part in the decision process about the duration of the antibiotic prescribed. Similarly, we did not find large differences in antibiotic prescriptions between children and adults. This provides further support for the argument that treatment durations are not increased because individual patient factors indicated a clinical need for prolonged treatment.

We only assessed whether the durations of antibiotic prescriptions followed guidelines and not whether antibiotics should have been completely avoided. Previous work showed that a substantial proportion of prescriptions for acute cough and bronchitis, acute otitis media, acute sinusitis, acute sore throat, and impetigo in primary care are unnecessary.^11^ Therefore, for these conditions the total number of days of unnecessary antibiotic use will be higher than estimated here. For example, if 77% of antibiotic prescriptions for acute sinusitis were inappropriate,^11^ the percentage of days of antibiotic treatment not following guidelines would increase from 7.1% to 81.1% for this indication. Antibiotics might also be unnecessary for community acquired pneumonia, for which it has been estimated that up to 25% of cases are caused by viruses without a bacterial coinfection.^48^ However, it can be difficult to distinguish between patients with community acquired pneumonia requiring antibiotics and those who can safely be treated without antibiotics in the primary care setting. Similarly, in suspected urinary tract infection, significant bacteriuria (>10^3^) colony forming unit (CFU)/mL has been found in only 25-65% of patients aged less than 65 years given antibiotic treatment. In older adults, asymptomatic bacteriuria is common and does not require antibiotic treatment.^11^ ^49^ ^50^

For two of the antibiotic indications we studied, treatment was frequently prescribed for shorter durations than recommended—both urinary tract infections in men: acute cystitis and acute prostatitis. This could arise because prescribers appreciate that recommendations for these conditions are based on expert opinion and historical precedent rather than evidence, and that treatment durations for urinary tract infections in women have declined considerably in recent years. It needs to be established whether shorter treatment durations are effective for men with urinary tract infections, as this is a small but important patient population with a risk of harm from under-treatment. Notably, the conditions for which course durations tended to be less than recommended in the guidelines were those for which evidence supporting duration of treatment is weak (supplementary figs S5-S7).

In general, the preference seemed to be for antibiotic prescriptions with durations of five or seven days or multiples thereof, without a clear evidence base for this preference. The preference for such durations has been observed previously in other settings and depended more on local practice and subspecialty than on clinical features such as fever, comorbidities, and severity.^51^ ^52^ ^53^ In addition, for conditions where guidelines recommend longer durations, the percentage of prescriptions beyond the recommendation is substantially lower than for conditions where guidelines recommend a relatively short duration.

### Limitations of this study

One important limitation is that some antibiotic prescriptions may have been falsely linked to certain conditions in our study, because there is no automatic link between prescriptions and diagnoses in THIN.^29^ Results were, however, similar when restricting the analyses to only antibiotics mentioned in the guidelines for each specific condition, which increases the chance that the antibiotic was being used for the condition of interest. Although we excluded complicated, recurrent infections that may require longer treatment, we were not able to account fully for patient factors that might underlie decisions to prolong treatment. We were also not able to explore clinician factors that could underlie deviation from recommended durations of antibiotic treatment. For some patients in some cases longer durations would be appropriate; however, no clear evidence exists about the percentage of patients who should legitimately receive longer prescriptions. Moreover, for some infections, such as acute sinusitis, recent evidence indicates that the duration of antibiotic treatment should be shorter instead of longer than the recommended durations in the PHE 2013 guidance (five and seven days, respectively).^30^

We were unable to account for the possibility that treatment durations for some patients are influenced by pack sizes, which may include more than the prescriber would like dispensed, or by protocolised durations set in electronic prescribing decisions. Pack size could result in unintended antibiotic overuse, whereas electronic based decisions might be expected to guide prescribers to recommended durations. Both, however, are system factors that could be addressed to help prescribers make more patient tailored decisions about minimum effective duration of treatment.

The evidence base for optimal durations of treatment for respiratory tract infections in primary care is relatively small^27^ ^28^ ^54^; although there is increasing evidence that lengths of antibiotic courses can safely be reduced for several conditions in primary care,^18^ ^27^ clinicians may lack confidence in guidelines around duration of antibiotic treatment.^55^ ^56^ We were only able to measure prescribed antibiotics and cannot determine how often patients did not take or complete their prescribed course, previously estimated to be between one in 10 and one in four patients.^57^ ^58^ We did not exclude or separately analyse acute cystitis among pregnant women, for whom guidelines recommend seven days rather than three days.^31^ However, because women are pregnant for a minority of their lifespan, only a small proportion of the total number of cases of acute cystitis occur among pregnant women, thereby limiting the impact on the overall estimates.

### Conclusion and implications

Although previous work has shown that antibiotic use can potentially be substantially reduced by not prescribing antibiotics when they are unnecessary,^1^ ^11^ this study shows that unnecessary exposure to antibiotics may also be substantially reduced by aligning the course length more with guidelines and best available evidence. Highlighting the magnitude of this issue is only a first step. We need a better understanding of why clinicians tend to prescribe antibiotic courses that are longer than guideline recommendations, especially for respiratory tract infections. Poor guideline adherence may result from several factors, including lack of awareness and scepticism of specific guideline recommendations.^55^ ^56^ Moreover, the idea that shorter courses increase the risk of antibiotic resistance for common bacterial infections is not evidence based, but could—together with concerns about treatment failure due to undertreatment—make primary care doctors hesitant to prescribe shorter antibiotic courses in line with guidance. Understanding the reasons would enable the development of interventions and support tools that could increase adherence to the guidelines and reduce unnecessary exposure to antibiotics.

Overall, substantial reductions in antibiotic exposure can be accomplished by aligning duration of antibiotic prescriptions with guidelines.

**What is already known on this topic**

A clear causal link exists between antibiotic exposure and antibiotic resistance
Strategies to reduce antibiotic use in primary care focus on decisions to start treatment
It is not known to what extent excessive treatment duration contributes to antibiotic overuse in primary care

**What this study adds**

For many common infections treated in primary care, a substantial proportion of antibiotic prescriptions have durations that exceed those recommended in guidelines
Rates of treatment duration beyond guidelines are highest for respiratory tract infections and are similar among patients with and without comorbidities
Substantial reductions in antibiotic use in primary care could be achieved by closer compliance with recommended treatment durations

## Web extra

Extra material supplied by authors

## Notes

Contributors: KBP designed the study, cleaned and analysed the data, and drafted and revised the manuscript. He is the guarantor. SH and MJL contributed to the interpretation of data and revised the draft manuscript. ASW contributed to the statistical analysis plan and revised the draft manuscript. CAMM and JVR contributed to the design of the study and revised the draft manuscript. All authors approved the final version of the manuscript and agreed to be accountable for all aspects of the work. The corresponding author attests that all listed authors meet authorship criteria and that no others meeting the criteria have been omitted.

Ethical approval: The Health Improvement Network (THIN) data were used for this work. The data collection scheme for THIN is approved by the UK Multicentre Research Ethics Committee (reference No 07H1102103). In accordance with this approval, the study protocol was reviewed and approved by an independent Scientific Review Committee (reference No 16THIN071, 16THIN071-A1, and 16THIN071-A3).

Data sharing: THIN data were analysed under licence and are not available for sharing.

Transparency: The guarantor (KBP) affirms that the manuscript is an honest, accurate, and transparent account of the study being reported; that no important aspects of the study have been omitted; and that any discrepancies from the study as planned (and, if relevant, registered) have been explained.
