# Supplementary material for: Duration of antibiotic treatment for common infections in English primary care: cross sectional analysis and comparison with guidelines
Source: BMJ. 2019 Feb 27;364:l440. doi: 10.1136/bmj.l440 (PMC6391655; doi:10.1136/bmj.l440)
Supplement: Supplementary file 1 — Supplementary information: Additional tables, figures, and information [file pouk046953.ww1.pdf]

Supplementary file 1. Antibiotic treatment durations recommended in English guidelines.

Table S1 Antibiotic treatment durations recommended in English guidelines provided by Public Health England (PHE), National Institute for Health and Care Excellence (NICE) and Clinical Knowledge Summaries (CKS).

**Indication**	**PHE recommendation if antibiotic indicated between 2013 -15**[a]	**PHE recommendation if antibiotic indicated (2017)**	**Most recent NICE/PHE or CKS recommendation if antibiotic indicated**[b]
Acute sinusitis	*First line:*<br>Amoxicillin 7 days<br>Penicillin V 7 days<br>Doxycycline 7 days<br>*For persistent symptoms:*<br>Co-amoxiclav 7 days	*First line for delayed:*<br>Penicillin V 5 days<br>*Penicillin allergy or intolerance:*<br>Doxycycline 5 days<br>Clarithromycin 5 days<br>*Very unwell or worsening:*<br>Co-amoxiclav 5 days	*First choice:*<br>Penicillin V 5 days<br>*Penicillin allergy or intolerance:*<br>Doxycycline 5 days<br>Clarithromycin 5 days<br>Erythromycin (in pregnancy) 5 days<br>*Very unwell or worsening:*<br>Co-amoxiclav 5 days
Acute sore throat	*First choice:*<br>Penicillin V 10 days<br>*Penicillin allergy:*<br>Clarithromycin 5 days	*First choice:*<br>Penicillin V 5-10 days<br>*Penicillin allergy:*<br>Clarithromycin 5 days<br>*Penicillin allergy in pregnancy:*	*First choice:*<br>Penicillin V 5-10 days<br>*Penicillin allergy or intolerance:*<br>Clarithromycin 5 days<br>Erythromycin (in pregnancy) 5 days

		Erythromycin 5 days	
Acute bronchitis / cough	Amoxicillin 5 days<br><br>Doxycycline 5 days	*First choice:*<br>Amoxicillin 5 days<br><br>*Penicillin allergy:*<br>Doxycycline 5 days	*First choice:*<br>Amoxicillin 5 days<br><br>*Penicillin allergy:*<br>Doxycycline 5 days<br><br>*If amoxicillin and doxycycline contraindicated:*<br>Clarithromycin 5 days
Community-acquired pneumonia	*CRB65=0:*<br>Amoxicillin 7 days<br>Clarithromycin 7 days<br>Doxycycline 7 days<br><br>*CRB65=1-2:*<br>Amoxicillin + clarithromycin 7-10 days<br>Doxycycline 7-10 days	*CRB65=0:*<br>Amoxicillin 5 days*<br>Clarithromycin 5 days*<br>Doxycycline 5 days*<br><br>*CRB65=1-2:*<br>Amoxicillin + clarithromycin 7-10 days<br>Doxycycline 7-10 days	*CRB65=0:*<br>Amoxicillin 5 days*<br><br>*CRB65=0 & penicillin allergy*<br>Doxycycline 5 days*<br>Clarithromycin 5 days*<br><br>*CRB65=1-2:*<br>Amoxicillin + clarithromycin 7-10 days<br>Doxycycline 7-10 days

		* note comment 'review at 3 days, 7-10 if poor response'	* note comment 'review at 3 days, 7-10 if poor response'
Acute exacerbation of COPD	*First choice:*<br><br>Amoxicillin 5 days<br><br>Doxycycline 5 days<br><br>Clarithromycin 5 days<br><br>*If at risk of resistance:*<br><br>Co-amoxiclav 5 days	*First choice:*<br><br>Amoxicillin 5 days<br><br>Doxycycline 5 days<br><br>Clarithromycin 5 days<br><br>*If at risk of resistance:*<br><br>Co-amoxiclav 5 days	*First choice:*<br><br>Amoxicillin 5 days<br><br>Doxycycline 5 days<br><br>Clarithromycin 5 days<br><br>*Use alternative first choice (from a different class) when no improvement in symptoms on first choice taken for at least 2-3 days*<br><br>*High risk of treatment failure:*<br><br>Co-amoxiclav 5 days<br><br>Levofloxacin 5 days<br><br>Co-trimoxazole 5 days
Acute otitis media	*First choice:*<br><br>Amoxicillin 5 days<br><br>*Penicillin allergy:*<br><br>Erythromycin 5 days	*First choice:*<br><br>Amoxicillin 5 days<br><br>*Penicillin allergy:*<br><br>Erythromycin 5 days<br><br>Clarithromycin 5 days	*First choice:*<br><br>Amoxicillin 5-7 days<br><br>*Penicillin allergy or intolerance:*<br><br>Clarithromycin 5-7 days<br><br>Erythromycin 5-7 days

			Worsening symptoms on first choice taken for at least 2-3 days:<br><br>Co-amoxiclav 5-7 days
Acute cystitis non-pregnant females	Trimethoprim 3 days<br><br>Nitrofurantoin 3 days<br><br>*If nitrofurantoin and trimethoprim unsuitable and organism susceptible:*<br><br>Amoxicillin 3 days<br><br>*If high resistance risk:*<br><br>Fosfomycin 1 day	Nitrofurantoin 3 days<br><br>*If low risk or resistance:*<br><br>Trimethoprim 3 days<br><br>*If nitrofurantoin and trimethoprim unsuitable:*<br><br>Pivmecillinam 3 days<br><br>*If nitrofurantoin and trimethoprim unsuitable and organism susceptible:*<br><br>Amoxicillin 3 days<br><br>*If high resistance risk:*<br><br>Fosfomycin 1 day	Nitrofurantoin 3 days<br><br>*If low risk of resistance:*<br><br>Trimethoprim 3 days<br><br>*When no improvement in lower UTI symptoms on first-choice taken for at least 48 hours, or If nitrofurantoin and trimethoprim unsuitable:*<br><br>Pivmecillinam 3 days<br><br>Fosfomycin 1 day
Acute cystitis males	Trimethoprim 7 days<br><br>Nitrofurantoin 7 days	Nitrofurantoin 7 days (if fever, use alternative)	Trimethoprim 7 days<br><br>Nitrofurantoin 7 days

	If nitrofurantoin and trimethoprim unsuitable and organism susceptible:<br><br>Amoxicillin 7 days<br><br>*If high resistance risk:*<br><br>Fosfomycin 2 days	*If low risk or resistance:*<br><br>Trimethoprim 7 days<br><br>*If nitrofurantoin and trimethoprim unsuitable:*<br><br>Pivmecillinam 7 days<br><br>*If nitrofurantoin and trimethoprim unsuitable and organism susceptible:*<br><br>Amoxicillin 7 days<br><br>*If high resistance risk:*<br><br>Fosfomycin 2 days	
Acute prostatitis	*First choice:*<br><br>Ciprofloxacin 28 days<br><br>Ofloxacin 28 days<br><br>*Second line:*<br><br>Trimethoprim 28 days	*First choice:*<br><br>Ciprofloxacin 28 days<br><br>Ofloxacin 28 days<br><br>*Second line:*<br><br>Trimethoprim 28 days	*First choice:*<br><br>Ciprofloxacin 14 days*<br><br>Ofloxacin 14 days*<br><br>*If quinolone not suitable:*<br><br>Trimethoprim 14 days*<br><br>*Second choice, after discussion with specialist:*<br><br>Levofloxacin 14 days*<br><br>Co-trimoxazole 14 days*

			* Review treatment after 14 days and either stop the antibiotic or continue for a further 14 days if needed
Pyelonephritis	Ciprofloxacin 7 days<br>Co-amoxiclav 14 days	Ciprofloxacin 7 days<br>Co-amoxiclav 7 days<br>*If organism sensitive:*<br>Trimethoprim 14 days	Cefalexin 7-10 days<br>Co-amoxiclav 7-10 days<br>Ciprofloxacin 7 days<br>Trimethoprim 14 days<br>*If pregnant:*<br>Cefalexin 7 days<br>*Patients under 16 years:*<br>Cefalexin 7-10 days<br>Co-amoxiclav 7-10 days
Cellulitis	Flucloxacillin 7 days* and continue for further 7 days if slow response<br>*Penicillin allergy:*<br>Clarithromycin 7 days*<br>Clindamycin 7 days*<br>*Facial:*<br>Co-amoxiclav 7 days*<br>* If slow response, continue for further 7 days	Flucloxacillin 7 days* and continue for further 7 days if slow response<br>*Penicillin allergy:*<br>Clarithromycin 7 days*<br>*Penicillin allergy and taking statins:*<br>Doxycycline 7 days*	Flucloxacillin 7 days<br>*Penicillin allergy:*<br>Clarithromycin 7 days<br>*Mild facial cellulitis:*<br>Co-amoxiclav 7 days<br>*Mild facial cellulitis and penicillin allergy:*<br>*Clarithromycin 7 days*

		Unresolving:<br><br>Clindamycin 7 days*<br><br>*Facial (non-dental):*<br><br>Co-amoxiclav 7 days*<br><br>* If slow response, continue for further 7 days	*Adults with known lymphoedema, not requiring admission:*<br><br>Amoxicillin ≥14 days beyond time of observed definite clinical response;<br><br>Amoxicillin + flucloxacillin ≥14 days beyond time of observed definite clinical response;<br><br>*Adults with known lymphoedema, not requiring admission and penicillin allergy:*<br><br>Clarithromycin ≥14 days beyond time of observed definite clinical response<br><br>*Cellulitis in child suffering from varicella:*<br><br>Amoxicillin + flucloxacillin 7 days<br><br>*Cellulitis in child suffering from varicella and penicillin allergy:*<br><br>Ciprofloxacin + erythromycin or clarithromycin 7 days
Impetigo	*Extensive, severe, or bullous:*<br><br>Flucloxacillin 7 days<br><br>Clarithromycin 7 days	*Extensive, severe, or bullous:*<br><br>Flucloxacillin 7 days<br><br>Clarithromycin 7 days	*Extensive, systemic symptoms, or bullous:*<br><br>Flucloxacillin 7 days<br><br>*Penicillin allergy:*<br><br>Clarithromycin 7 days

			Erythromycin 7 days
Scarlet fever	Not in guidance	Penicillin V 10 days<br><br>*Penicillin allergy:*<br>Clarithromycin 5 days	Penicillin V 10 days<br><br>*If penicillin V unsuitable:*<br>Amoxicillin 10 days<br><br>*Penicillin allergy:*<br>Azithromycin 5 days
Gastroenteritis	*If systemically unwell and campylobacter suspected:*<br>Clarithromycin 5-7 days	*If systemically unwell and campylobacter suspected and treated within 3 days:*<br>Clarithromycin 5-7 days	*Campylobacter positive and one of the following: severe or worsening symptoms, immunocompromised, symptoms lasted longer than 1 week:*<br>Erythromycin 5-7 days<br><br>*If erythromycin not tolerated:*<br>Clarithromycin 5-7 days<br>Azithromycin 5-7 days<br>Ciprofloxacin 5-7 days

[a]*PHE guideline recommendations remained the same during the study period (2013-2015).*

[b]*NICE 2017 guidance has been developed in collaboration with PHE*

Supplementary files 2:

Multiple imputation

Although the quantity of antibiotics prescribed was available for virtually all prescriptions (>99%) the daily dose and hence duration was missing between 10-20% of the prescriptions, dependent on the condition. Multiple imputation via chained equations using sequential regression trees were used to impute these missing durations.[38] This non-parametric method can capture potential complex interactions and non-linear relationships in the underlying data and can result in more plausible imputations in complex settings than standard sequential regression imputation techniques.[38] For all indications durations were imputed based on practice identifier, age, antibiotic group (different groups for each condition based on the first 5 characters of the Anatomical Therapeutic Chemical (ATC) code and the number of observations, except for azithromycin, which was used as a separate group given its long half-life) and the quantity of antibiotics being prescribed. Regarding the group of antibiotics, separate categories were used as long as there were at least 100 observations with that antibiotic group. The remaining antibiotics were grouped in the 'other antibiotic' category.

In addition, we evaluated whether one or more of the following potential predictors of antibiotic duration should be included in the imputation models as well using models restricted to complete cases: sex, chronic kidney disease, chronic respiratory disease, asthma, coronary heart disease, immunosuppressive disease, use of immunosuppressive drugs, use of systemic corticosteroids, or use of inhaled corticosteroids. We included these additional variables in imputation models if they had $p<0.01$ in the multivariable model. In total, 10 datasets were imputed for each condition. Although there was some variation in the imputed value for individual prescriptions, the summary estimates for each imputed dataset were virtually identical and therefore 10 imputations was judged sufficient.

The resulting imputations were almost solely driven by the combination of the antibiotic groups, age and the total quantity prescribed, e.g. the number of tablets. Occasionally some other variables, such as chronic kidney disease were associated with the treatment duration, however this had a negligible influence on the summary estimates for each imputed dataset.

Supplementary file 3: Additional figures and results of additional analyses.

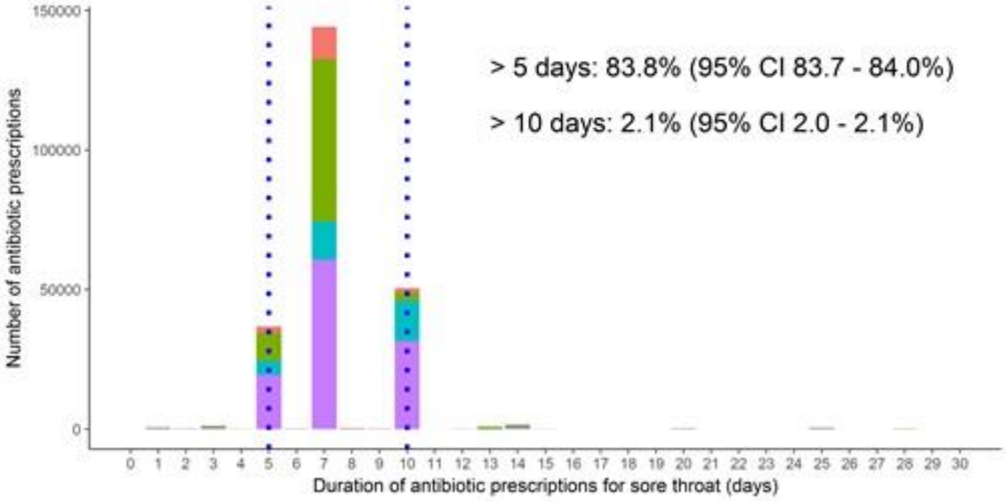

Fig S1 Durations of antibiotic prescriptions for acute sore throat. The purple portions of the bars are observed data for penicillin V, the blue portions are imputed data for penicillin V, the green portions are observed data for other antibiotics, and the red portions are imputed data for other antibiotics. The dark blue dotted vertical lines represent the main analysis using durations recommended by 2013 PHE guidance. That guidance recommends 10 days for penicillin V and 5 days for clarithromycin.

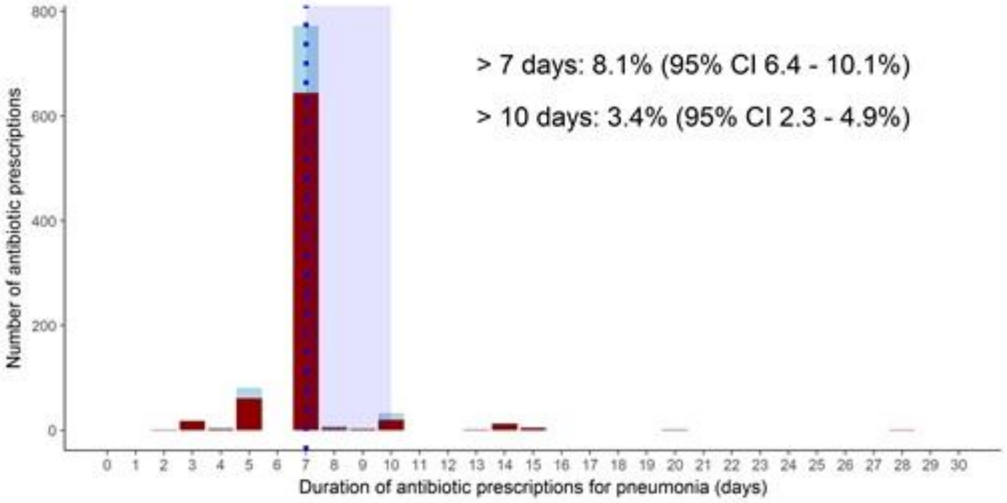

Fig S2 Durations of antibiotic prescriptions for community-acquired pneumonia. The dark red portions of the bars are observed data, while the light blue portions are imputed data. The dotted vertical lines represent the duration recommended by 2013 PHE guidance: 7 days for patients with a CRB-65 score of 0. The shaded area represents the recommended duration (7-10 days) for patients with a higher CRB-65 score.

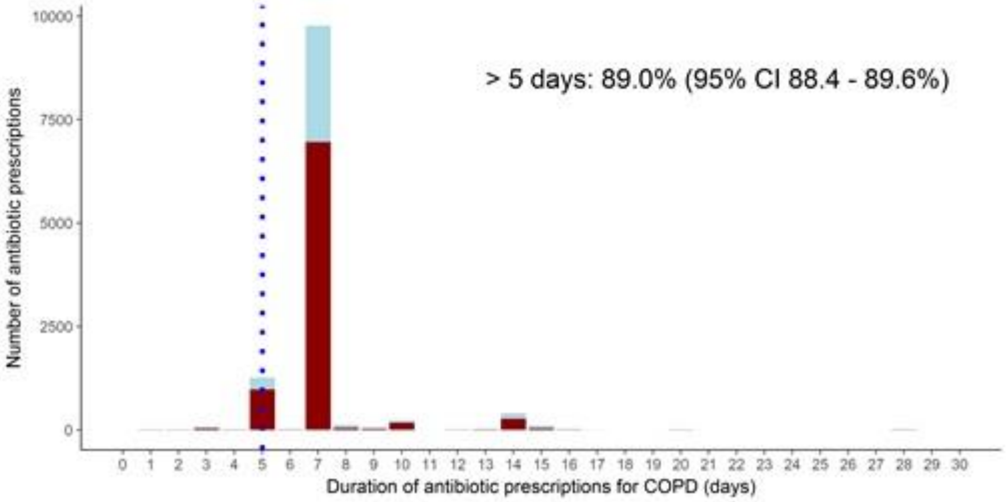

Fig S3 Durations of antibiotic prescriptions for acute exacerbations of COPD. The dark red portions of the bars are observed data, while the light blue portions are imputed data. The dotted vertical line represents the duration recommended by PHE guidance.

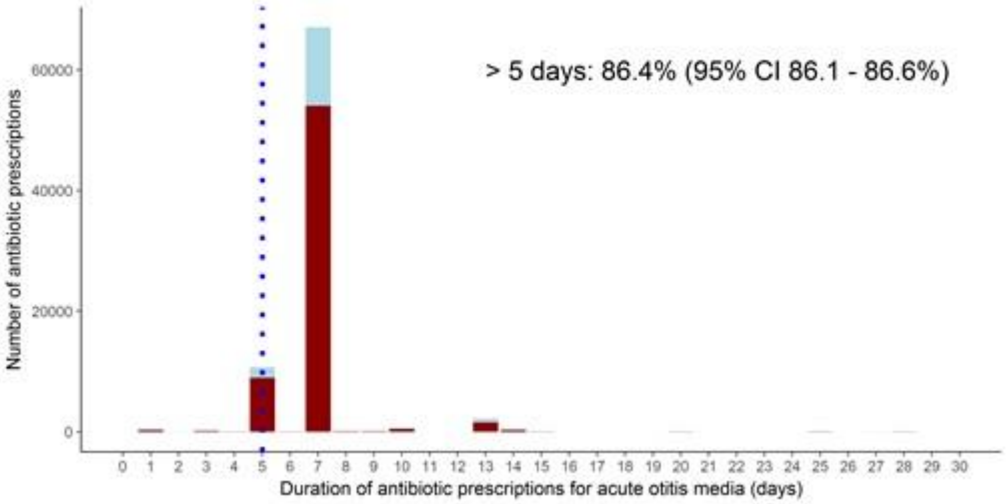

Fig S4 Durations of antibiotic prescriptions for acute otitis media. The dark red portions of the bars are observed data, while the light blue portions are imputed data. The dotted vertical line represents the duration recommended by PHE guidance.

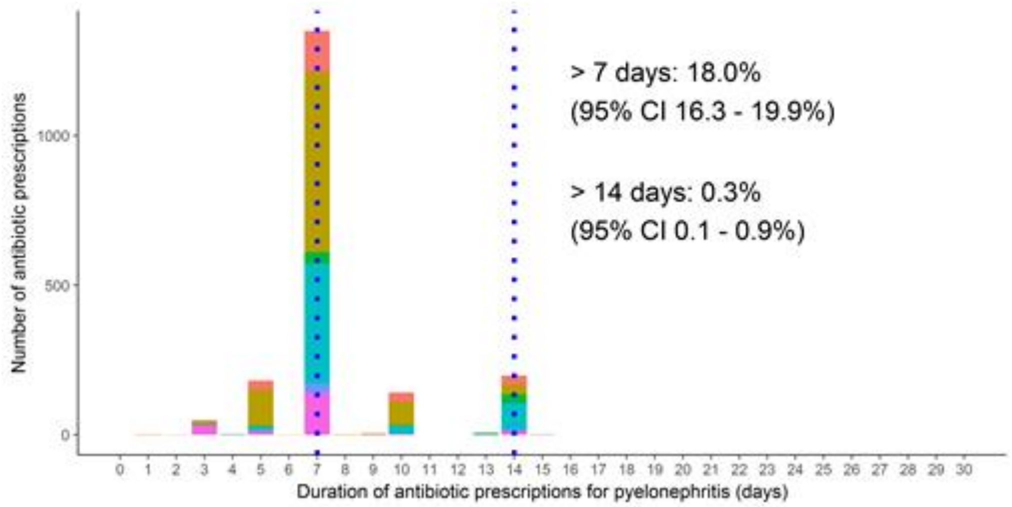

Fig S5 Durations of antibiotic prescriptions for pyelonephritis. The pink portions of the bars are observed data for trimethoprim, the purple portions are imputed data for trimethoprim, the blue portions are observed data for co-amoxiclav, the green portions are imputed data for co-amoxiclav, the other portions are observed data for other antibiotics, and the red portions are imputed data for other antibiotics. The dotted vertical line represents the duration recommended by 2013 PHE guidance for all antibiotics, except co-amoxiclav (trimethoprim in 2017 PHE guidance). The dashed vertical line represents the recommended duration for co-amoxiclav (during study period) and trimethoprim (2017 PHE guidance).

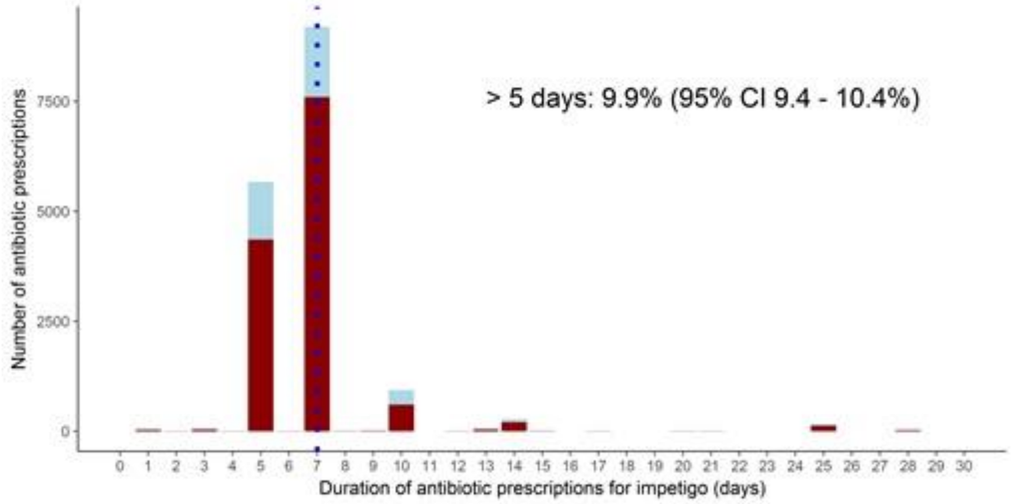

Fig S6 Durations of antibiotic prescriptions for impetigo. The dark red portions of the bars are observed data, while the light blue portions are imputed data. The dotted vertical line represents the duration recommended by English guidelines.

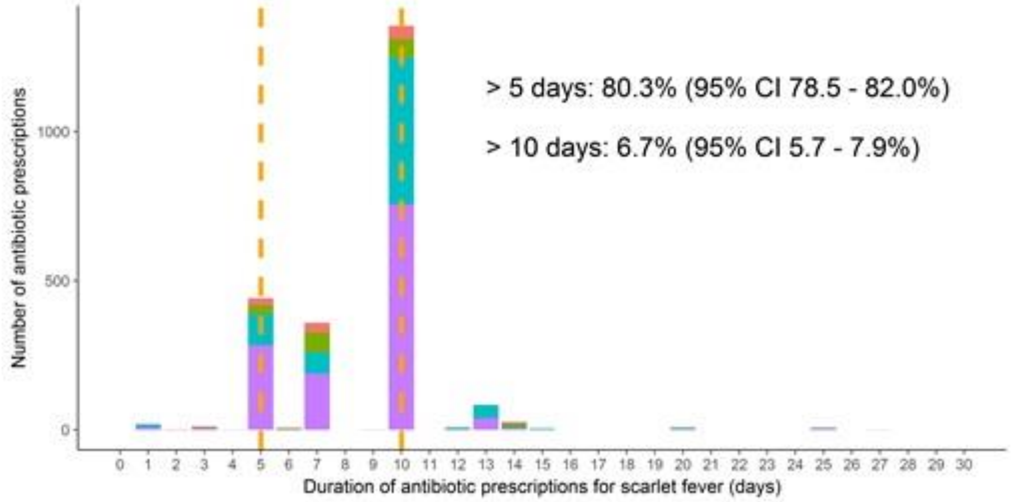

Fig S7 Durations of antibiotic prescriptions for scarlet fever. The purple portions of the bars are observed data for penicillin V and amoxicillin, the blue portions are imputed data for penicillin V and amoxicillin, the green portions are observed data for other antibiotics, and the red portions are imputed data for other antibiotics. The dashed orange vertical line represents the duration recommended by English guidelines (10 days for penicillin V and amoxicillin; 5 days for clarithromycin and azithromycin).

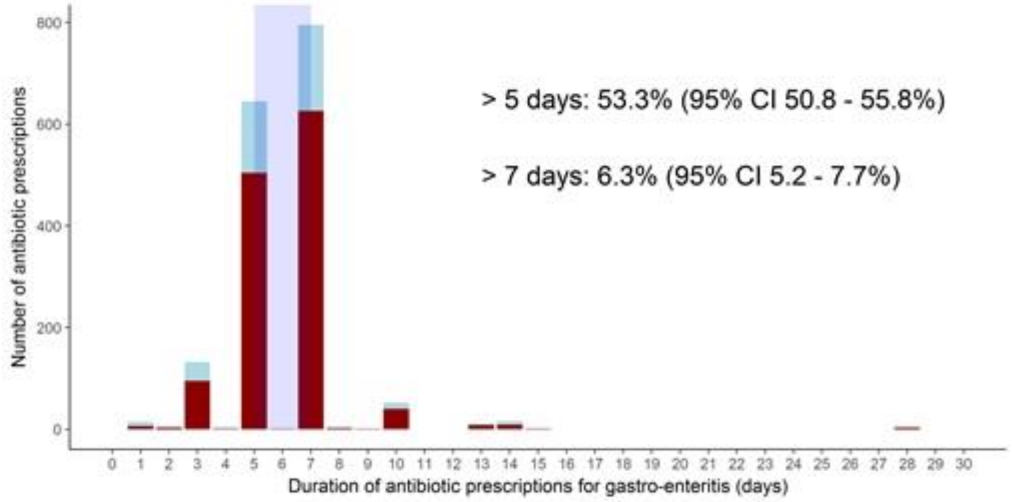

Fig S8 Durations of antibiotic prescriptions for acute gastroenteritis. The dark red portions of the bars are observed data, while the light blue portions are imputed data. The shaded are represents the duration recommended by English guidelines: 5-7 days of treatment if antibiotics are indicated.

Table S2 The percentage of antibiotics with a duration exceeding the guideline recommendations for different age-groups.

	**Age-category**	**Recommended duration (days)**	**Percentage of antibiotics with duration exceeding guideline recommendations**
Acute sinusitis	<16 y (n=2,028)	7	8.8 (95% CI 7.6 – 10.1)
	≥16 y (n=74,655)	7	9.7 (95% CI 9.4 – 9.9)
Acute sore throat	<16 y (n=89,131)	5	70.7 (95% CI 70.4 – 71.0)
		10	3.5 (95% CI 3.4 – 3.7)
	≥16 y (n=150,100	5	91.6 (95% CI 91.5 – 91.7)
		10	1.2 (95% CI 1.2 – 1.3)
Acute bronchitis / cough	<16 y (n=79,997)	5	89.0 (95% CI 88.7 – 89.2)
	≥16 y (n=306,975)	5	84.7 (95% CI 84.6 – 84.9)
Community-acquired pneumonia	<16 y (n=56)	5	91.8 (95% CI 80.8 – 96.7)
		7	8.7 (95% CI 3.1 – 22.4)
		10	5.7 (95% CI 1.7 – 17.6)
	≥16 y (n=896)	5	89.1 (95% CI 86.7 – 91.1)
		7	8.0 (95% CI 6.3 – 10.1)
		10	3.2 (95% CI 2.2 – 4.8)
Acute otitis media	<16 y (n=57,290)	5	89.9 (95% CI 89.6 – 90.2)
		7	7.3 (95% CI 7.1 – 7.5)
	≥16 y (n=25,764)	5	78.6 (95% CI 78.1 – 79.1)
		7	1.9 (95% CI 1.8 – 2.1)
Acute cystitis among females	16 – 49 y (n=23,820)	3	51.5 (95% CI 50.9 – 52.2)
	≥ 50 y (n=24,914	3	57.5 (95% CI 56.8 – 58.1)
Acute Prostatitis	<35 y (n=221)	14	56.0 (95% CI 49.3 – 62.5)
		28	12.4 (8.6 – 17.5)
	35-64 y (n=1,137)	14	57.4 (95% CI 54.5 – 60.2)

		28	13.4 (95% CI 11.5 – 15.5)
	≥ 65 y (n=480)	14	50.7 (95% CI 46.0 – 55.4)
		28	10.7 (95% CI 8.1 – 13.9)
Pyelonephritis	<16 y (n=53)	7	27.2 (95% CI 15.9 – 42.4)
		14	0
	≥16 y (n=1,895)	7	17.7 (95% CI 16.0 – 19.6)
		14	0.4 (95% CI 0.1 – 0.9)
Acute cellulitis	<16 y (n=2,554)	7	11.6 (95% CI 10.3 – 13.0)
		14	2.2 (95% CI 1.6 – 2.9)
	≥16 y (n=52,056)	7	14.1 (95% CI 13.8 – 14.4)
		14	0.7 (95% CI 0.6 – 0.8)
Impetigo	<16 y (n=10,247)	7	12.1 (95% CI 11.4 – 12.8)
	≥16 y (n=6,352)	7	6.4 (95% CI 5.8 – 7.0)
Scarlet fever	<16 y (n=2,205)	5	79.6 (95% CI 77.7 – 81.4)
		10	6.8 (95% CI 5.8 – 8.0)
	≥16 y (n=145)	5	90.5 (95% CI 84.2 – 94.4)
		10	4.8 (95% CI 2.2 – 10.2)
Gastroenteritis	<16 y (n=196)	5	70.7 (95% CI 62.4 – 77.7)
		7	16.5 (95% CI 11.0 – 23.9)
	≥16 y (n=1,505)	5	51.0 (95% CI 48.5 – 53.6)
		7	5.0 (95% CI 4.0 – 6.2)

Table S3 The percentage of antibiotics with a duration exceeding the guideline recommendations for antibiotics mentioned in the guidelines

	**Antibiotics (n, % of total antibiotic prescriptions for this condition)**	**Recommended duration (days)**	**Percentage of antibiotics with duration exceeding guideline recommendations**
Acute sinusitis	Amoxicillin, doxycycline, penicillin V, co-amoxiclav (n=66,992, 87.4%)	7	9.4% (95% CI 9.2 – 9.7%)
Acute sore throat	Penicillin V (n=148,001, 61.9%)	10	1.2% (95% CI 1.1 – 1.2%)
	Clarithromycin, erythromycin (n=38,057, 15.9%)	5	83.3% (95% CI 82.9 – 83.7%)
Acute bronchitis / cough	Amoxicillin, doxycycline(n=,304,610, 78.7%))	5	84.3% (95% CI 84.2 – 84.4%)
Community-acquired pneumonia	Amoxicillin, clarithromycin, doxycycline (n=719, 75.5%)	7	6.6% (95% CI 4.8 – 8.9%)
		10	2.5% (95% CI 1.5 – 4.2%)
Acute COPD exacerbation	Amoxicillin, doxycycline, clarithromycin, erythromycin, co-amoxiclav, (n=11,573, 95.9%)	5	89.4% (95% CI 88.8 – 90.0%)
Acute otitis media	Amoxicillin, clarithromycin, erythromycin (n=74,544, 89.8%)	5	88.3% (95% CI 88.0 – 88.5%)
Acute cystitis among females	Nitrofurantoin, trimethoprim, amoxicillin (n=44,357, 91.0%)	3	50.7% (95% CI 50.2 – 51.2%)

Acute Prostatitis	Ciprofloxacin, ofloxacin, trimethoprim (n=1,449, 78.8%)	28	10.4% (95% CI 8.9 – 12.1%)
Pyelonephritis	Ciprofloxacin, co-amoxiclav(n=1,300, 66.7%)	7	21.6% (95% CI 19.4 – 24.0%)
	Trimethoprim (n=242, 12.4%)	14	0.3% (95% CI 0.0 – 4.0%)
	Co-amoxiclav (n=614, 31.5%)	14	0.3% (95% CI 0.1 – 1.2%)
Acute cellulitis	Flucloxacillin, clarithromycin, clindamycin, co-amoxiclav, erythromycin (n=48,000, 87.9%)	7	12.9 (95% CI 12.6 – 13.2)
		14	0.5% (95% CI 0.4 – 0.6%)
Impetigo	Flucloxacillin, clarithromycin, erythromycin (n=14,844, 89.4%)	7	9.8% (95% CI 9.3 – 10.3%)
Scarlet fever	Clarithromycin, azythromycin (n=98, 4.2%)	5	85.5% (95% CI 72.8 – 92.9%)
	Penicillin V, amoxicillin (n=2,057, 87.5%)	10	6.5% (95% CI 5.4 – 7.7%)
Gastroenteritis	Clarithromycin, erythromycin(n=217, 12.8%)	5	82.8% (95% CI 77.0 – 87.3%)
		7	2.4% (95% CI 1.0 – 5.7%)

Table S4 The percentage of antibiotics with a duration exceeding the guideline recommendations when restricting the analysis to data without missing values.

	**Recommended treatment duration (days)**	**Percentage of antibiotics with duration exceeding recommendations (95% CI)**
Acute sinusitis (n=76,683)	7	7.7 (7.4 – 7.9)
Acute sore throat (n=239,231)	5[b]	83.6 (83.4 – 83.8)
	10[b]	2.0 (1.9 – 2.0)
Acute bronchitis / cough (n=386,972)	5	85.2 (85.1 – 85.3)
Community-acquired pneumonia (n=952)	7[c]	6.2 (4.7 – 8.0)
	10[c]	2.9 (1.8 – 4.0)
Acute COPD exacerbation (n=12,067)	5	88.1 (87.4 – 88.7)
Acute otitis media (n=83,054)	5	86.0 (85.7 – 86.2)
Acute cystitis females (n=48,734)	3	55.0 (54.6 – 55.5)
Acute cystitis males (n=4,276)	7	3.6 (2.9 – 4.2)
Acute prostatitis (n=1,838)	28	11.4 (9.9 – 13.1)
Pyelonephritis (n=1,948)	7[g]	15.8 (14.1 – 17.7)
	14[g]	0.3 (0.1 – 0.5)
Acute cellulitis (n=54,610)	7[h]	12.5 (12.2 – 12.8)
	14[h]	0.6 (0.5 – 0.7)
Impetigo (n=16,599)	7	8.4 (8.0 – 8.9)
Scarlet fever (n=2,350)	5[i]	77.8 (75.7 – 80.0)
	10[i]	5.7 (4.5 – 6.9)
Gastroenteritis (n=1,701)	5[j]	53.8 (51.1 – 56.3)
	7[j]	6.0 (4.7 – 7.3)

Table S5 The percentage of antibiotics with a duration exceeding the guideline recommendations using most recent guideline recommendation.

	**Recommended treatment duration (days)**	**Percentage of antibiotics with duration exceeding recommendations**	**95% CI**	**Excess days (% of total days)**
Acute sinusitis (n=76,683)	5	89.2	89.0 – 89.4	176,136 (31.5)
Acute sore throat (n=239,231)	5	83.8	83.7 – 84.0	640,381 (35.0)
	10	2.1	2.0 – 2.1	72,001 (3.9)
Acute bronchitis / cough (n=386,972)	5	85.6	85.5 – 85.7	805,051 (29.5)
Community-acquired pneumonia (n=952)	5	89.2	86.9 – 91.2	2,443 (34.1)
	7	8.1	6.4 – 10.1	744 (10.3)
	10	3.4	2.3 – 4.9	535 (7.4)
Acute COPD exacerbation (n=12,067)	5	89.0	88.4 – 89.6	26,732 (30.8)
Acute otitis media (n=83,054)	5	86.4	86.1 – 86.6	193,262 (31.9)
	7	5.6	5.5 – 5.8	50,163 (8.3)
Acute cystitis females (n=48,734)	3	54.6	54.1 – 55.0	99,321 (40.5)
Acute cystitis males (n=4,276)	7	4.2	3.6 – 4.9	1541 (5.6)
Acute prostatitis (n=1,838)	14	55.5	53.2 – 57.7	15,838 (41.3)
Pyelonephritis (n=1,948)	7	18.0	16.3 – 19.9	2,135 (14.1)
	14	0.3	0.1 – 0.9	269 (1.8)
Acute cellulitis (n=54,610)	7	13.9	13.6 – 14.2	47,063 (11.2)
	14	0.8	0.7 – 0.9	6,719 (1.6)
Impetigo (n=16,599)	7	9.9	9.4 – 10.4	13,948 (11.8)

Scarlet fever (n=2,350)	5	80.3	78.5 – 82.0	9,808 (45.7)
	10	6.7	5.7 – 7.9	1,493 (7.0)
Gastroenteritis (n=1,701)	5	53.3	50.8 – 55.8	3,071 (27.3)
	7	6.3	5.2 – 7.7	1,260 (11.2)
